# 

**Published:** 1873-09

**Authors:** 


### Contents of September Number.

### ORIGINAL DEPARTMENT.

ART. I.—A Paper read before the Montgomery County Medical Society, Ill. June, 1873. By T. D. Washburn, M. D. .......... 41
ART. II.—A Report of Five Cases of Colles Fracture, treated after the plan of Prof. E. M. Moore. By B. L. Hovey, M. D. .......... 47
ART. III.—Rubber Bands as aids in Stethoscopic Auscultation. By J. W. Southworth, M. D. .......... 49
ART. IV.—A Case of Renal Tumor. By Dr. Denman .......... 50

### MISCELLANEOUS.

Ovarian Tumor Removed by Enucleation .......... 52
The Treatment of Nervous Diseases in Syphilitic Patients .......... 55
Tatooing the Cornea .......... 57
The Controversy on Septicæmia .......... 58
Infantile Paralysis .......... 59
Carbonate of Amonia in Scarlet Fever .......... 60
The Pathology and Diagnosis of Acute Bright's Disease .......... 62
Atropine as an Antidote to Opium .......... 67
On the Treatment of Chronic Dysentery .......... 70

### EDITORIAL DEPARTMENT.

Laws of New York Establishing and Regulating the Office of Coroner... 71
Is Niagara River Water Healthy? .......... 72
Items and Remarks. By E. N. Brush .......... 72
Errata .......... 75

Books Reviewed:

A Guide to Urinary Analysis by Henry G. Piffard, M. D. .......... 76
The Principles and Practice of Surgery. By Frank H. Hamilton, M. D. .......... 76
Text-Book of Physiology. General, Special and Practical. By John Hughes Bennett, M. D. .......... 77
Manual of Chemical Analysis as applied to Examination of Medicinal Chemicals. By F. Hoffman, Ph. D. .......... 77
Contributions to Practical Surgery. By Geo. W. Norris, M. D. .......... 78
Chemistry, General, Medical, and Pharmaceutical. By John Attfield, Ph. D., F. C. S. .......... 79
Skin Diseases, their Pathology Diagnosis and Treatment. By Tilbury Fox, M. D. .......... 79

Books and Pamphlets Received .......... 80

